# Profiling Complement System Components in Primary CNS Vasculitis

**DOI:** 10.3390/cells10051139

**Published:** 2021-05-08

**Authors:** Milani Deb-Chatterji, Christian W. Keller, Simon Koch, Heinz Wiendl, Christian Gerloff, Tim Magnus, Jan D. Lünemann

**Affiliations:** 1 Department of Neurology, University Medical Center Hamburg-Eppendorf, 20246 Hamburg, Germany; m.deb-chatterji@uke.de (M.D.-C.); si.koch@uke.de (S.K.); gerloff@uke.de (C.G.); t.magnus@uke.de (T.M.); 2 Department of Neurology with Institute of Translational Neurology, University Hospital Münster, 48149 Münster, Germany; Christianwolfgang.Keller@ukmuenster.de (C.W.K.); heinz.wiendl@ukmuenster.de (H.W.)

**Keywords:** complement system, vasculitis, PACNS, CSF, neuroinflammation

## Abstract

Complement activation has been implicated in the pathogenesis of many vasculitic syndromes such as anti-neutrophil cytoplasmic antibody (ANCA)-associated vasculitides. Using an array-based multiplex system, we simultaneously quantified serum and CSF levels of activated and regulatory complement system proteins in patients with primary CNS vasculitis (PACNS; *n* = 20) compared to patients with non-inflammatory conditions (*n* = 16). Compared to non-inflammatory controls, levels of C3a, C5a, and SC5b-9, indicative for general activation of the complement system, of C4a, specific for the activation of the classical pathway, Ba and Bb, reflective for alternative complement activation as well as concentrations of complement-inhibitory proteins factor H and factor I were unchanged in patients with PACNS. Our study does not support the hypothesis that complement activation is systemically increased in patients with PACNS.

## 1. Introduction

Primary angiitis of the central nervous system (PACNS) is a severe inflammatory disease affecting medium or small vessels of the CNS and is an important differential diagnosis in stroke of young adults. Its pathogenesis is poorly understood and discrimination of PACNS from its mimics such as reversible cerebral vasoconstriction syndrome (RCVS) or moyamoya disease (MMD) remains challenging. A recent exploratory proteomic study identified a significant enrichment of complement pathway proteins in the cerebrospinal fluid (CSF) of patients with PACNS compared to patients with non-inflammatory neurological diseases (NIND) and controls with RCVS [1], indicating that sustained complement activation contributes to PACNS pathology. Indeed, hyperactivation of the complement system associated with severe inflammatory responses in numerous organs including the CNS is frequently observed in several autoimmune diseases or in subjects with dysfunctional complement regulatory proteins. Here, we systematically profiled complement activation pathways in a large cohort of patients with PACNS compared with patients with NIND.

## 2. Materials and Methods

### 2.1. Study Subjects

A total number of 36 individuals from the University Medical Center Hamburg-Eppendorf, Hamburg, Germany, were included in the study. Twenty patients suffered from PACNS. Patients were considered to have an active course of disease (*n* = 18/20) when they were newly diagnosed or had a relapse, and to be in remission (*n* = 2/20) at time of blood sampling when they either had no clinical symptoms or were clinically stable under a successful immunosuppressive treatment. In addition, we included 16 patients with NIND, demographically matched with our PACNS cohort (age, median and IQR: 44.5 (33.75–44.5) for PACNS and 59.5 (33.25–66.5) for NIND; female to male ratio: 10/10 for PACNS and 9/7 for NIND). Patients with NIND suffered from normal pressure hydrocephalus (*n* = 7), dementia (*n* = 1), psychosomatic/psychiatric disease (*n* = 2), migraine (*n* = 2), intracranial aneurysm (*n* = 2) or cardiac syncope (*n* = 1), peroneal nerve palsy (*n* = 1) at the time of CSF and blood sampling. The study was approved by the local ethics committee, following the tenets of the Declaration of Helsinki. Written informed consent was obtained from all participants.

### 2.2. Complement Profiling

Upon venipuncture, samples were held at room temperature for 30 min to allow for clot retraction then centrifugation at 4 °C was performed and serum specimens were immediately frozen down at −80 °C. After lumbar puncture, CSF was centrifuged at room temperature and also immediately stored at −80 °C. While levels of complement proteins tend to be higher in serum as compared to plasma specimens, serum and plasma levels strongly correlate [2,3]. A multiplex ELISA based on chemiluminescence was used according to the manufacturer’s recommendations (Tecomedical AG, Sissach, Switzerland) to systematically profile complement proteins in both serum and CSF samples.

### 2.3. Statistics

Mann-Whitney test was performed to compare levels of complement proteins between clinical cohorts. GraphPad-Prism v7.0b was used for statistical analyses.

## 3. Results

CSF and serum complement activation was profiled in 20 patients with PACNS compared to 16 demographically matched patients with NIND (Table 1).

Patients with PACNS suffered from acute focal neurological deficits, encephalopathy, headache and/or seizures. Diagnosis was supported by brain MRI which revealed acute ischemic stroke or new/progressive contrast enhanced lesions with angiographic evidence for vessel irregularities in multiple intracranial vessels (17/20 patients) and/or by brain biopsy with documented transmural inflammation and vessel wall damage of small to medium-sized vessels (6/20 patients). Most patients with PACNS had active disease (18/20 patients), 10 of the active patients had disease onset, 8 had a clinical relapse, 2 patients were clinically stable without new imaging pathologies (Table S1). None of these patients suffered from any (chronic) pre-existing comorbidities, that might have allowed any doubts about the diagnosis, or received medication that might have an influence on the complement pathways.

**Figure 1:**
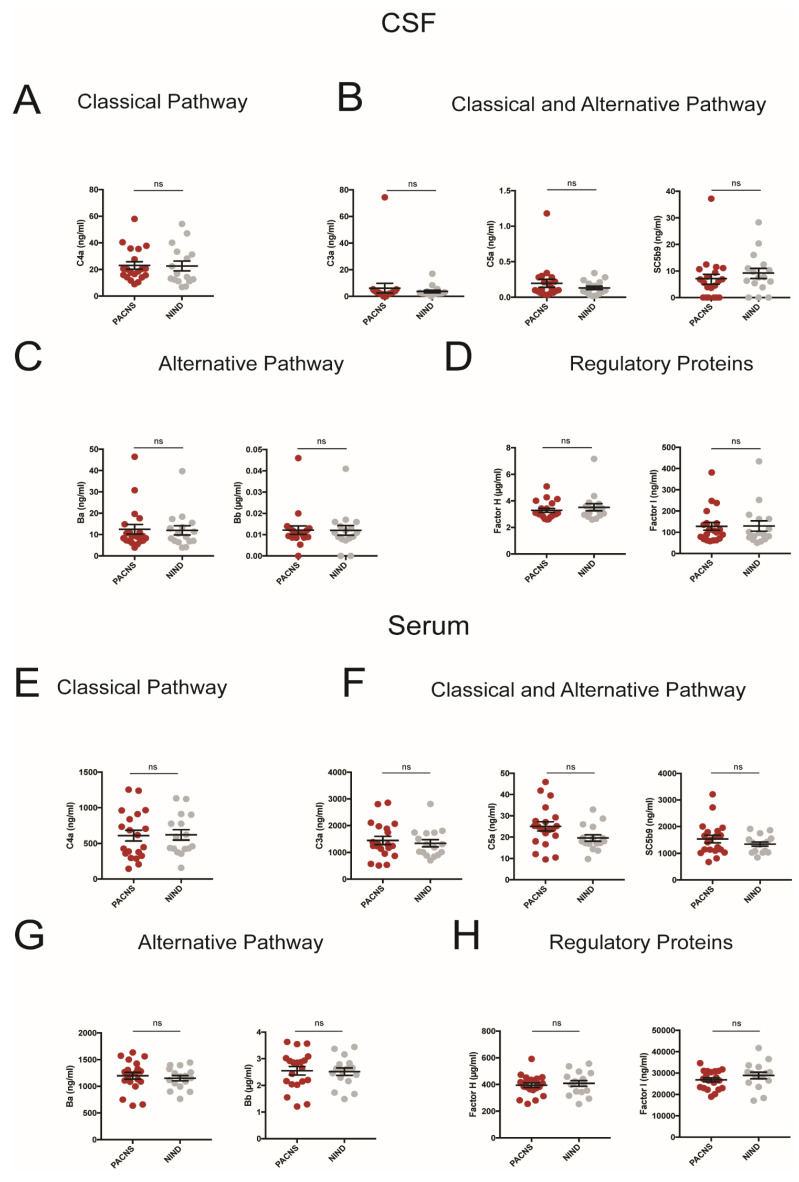
CSF (**A**–**D**) and Serum (**E**–**H**) concentrations of complement proteins representative for distinct complement activation pathways and regulatory pathways (**A**,**E**: classical pathway; **B**,**F**: classical and alternative pathway; **C**,**G**: alternative pathway; **D**,**H**: regulatory proteins). PACNS patients compared to patients with non-inflammatory neurologic disease (NIND). Each dot represents an individual patient.

Formation of the terminal complement complex, i.e., membrane attack complex, can be triggered by two separate proteolytic pathways, the classical pathway (homologous to the lectin pathway) and the alternative pathway. Signaling events from both pathways converge onto a common effector pathway, known as the terminal cascade, which enables lysis and phagocytosis of tagged target cells. Both CSF and serum levels of C3a, C5a, and the soluble terminal complement complex SC5b-9, indicative for general activation of the complement system, of C4a, specific for the activation of the classical pathway, Ba and Bb, reflective for alternative complement activation as well as concentrations of complement-inhibitory proteins factor H and factor I were unchanged in patients with PACNS as compared to patients with NIND (Figure 1). Within the PACNS cohort, we did not detect statistically significant differences between patients with and without immunotherapy or between biopsy-proven cases as compared to patients without biopsy.

## 4. Discussion and Conclusions

PACNS is a poorly understood inflammatory disease affecting the blood vessel walls in the brain, spinal cord and meninges in absence of systemic inflammation. The CSF circulates throughout these regions, shows abnormalities in the vast majority of PACNS patients, and its analysis can, therefore, not only support the diagnosis but also provide important information to better define mechanistic underpinnings of the disease [4]. Complement activation has been implicated in the pathogenesis of many vasculitic syndromes such as anti-neutrophil cytoplasmic antibody (ANCA)-associated vasculitides (AAV) and systemic inhibition of the complement system, particularly by targeting C5a, is a promising strategy for remission induction in AAV [5].

Using a mass spectrometry–based approach to characterize the CSF proteome in PACNS relative to non-inflammatory diseases, Mandel-Brehm and colleagues [1] identified several significantly dysregulated proteins in PACNS CSF that function within the complement activation pathway, specifically affecting the alternative and terminal cascade. Validation by western blot and ELISA was performed for one complement factor, uncleaved complement C5, its concentration was increased in the CSF in 5 out of 8 patients with PACNS [1].

Our approach to simultaneously determine 8 complement activation and regulatory proteins in a single specimen allowed us to systematically interrogate the complement system at high resolution and to profile its activation at the level of complement pathways instead of individual proteins. Since elevated levels of uncleaved complement proteins does not necessarily reflect complement activation, we determined the concentration of complement activation products in addition to regulatory proteins. Thus, the array of profiled complement components is different from the work by Mandel-Brehm [1].

While we did not find any evidence for enhanced complement activation in the CSF of patients with PACNS, our study has limitations which should be accounted for when interpreting the results. Only 6 out of 20 patients had biopsy-proven PACNS. However, complement activation profiles in biopsy-proven cases did not differ from those without a biopsy being taken and focusing only on biopsy-confirmed cases would have considerably limited available patient numbers and could have biased our study. Our study does not formally exclude that complement activation in PACNS occurs on the endothelium in situ without being detectable in the CSF. However, to the best of our knowledge, there is no histological data demonstrating complement-deposition or activation in PACNS endothelium.

In summary, our data do not support the hypothesis that enhanced or sustained activation of complement cascade pathways contribute to PACNS pathology or that CSF proteins produced during complement activation could be used as surrogate marker to aid the diagnosis of PACNS. Our study is complementary to the work of Mandel-Brehm et al. [1] and underlines the importance of both exploratory and validation studies in larger cohorts of patients with the ultimate goal of identifying targeted therapeutic interventions for this poorly understood disease.

## Figures and Tables

**Table 1:** Clinical, demographical characteristics and laboratory findings in patients with PACNS and NINDS.

Clinical, Demographical and Laboratory Characteristics	PACNS	NINDS
Age–median (IQR)	44.5 (33.75–44.5)	59.5 (33.25–66.5)
Male—*n* (%)	10/20 (50)	9 (56.3)
Immunosuppression at time of CSF/Blood sampling—*n* (%)	9/20 (45)	-
Brain Imaging		-
Angiographic abnormality—*n* (%)	16/20 (80)	-
Contrast-enhancement leptomeningeal/parenchymatous—*n* (%)	4/20 (20)	-
CSF parameters		-
WBC count, cells/µL—median (IQR)	5 (1.25–56.0)	3 (2–4.75)
Protein level, mg/L—median (IQR)	493.5 (362–607.5)	415.5 (353.75–542.5)
Oligoclonal bands, pos—*n* (%)	5/20 (25)	0
Intrathecal Ig-synthesis, yes—*n* (%)	7/20 (35)	0
Brain biopsy—*n* (%)	11/20 (55)	-
High-suspected—*n* (%)	15/20 (75)	-
Biopsy-proven—*n* (%)	6/20 (30)	-
Lymphocytic—*n* (%)	4/6 (66.6)	-
Granulomatous—*n* (%)	2/6 (33.3)	-
Necrotizing—*n* (%)	0 (0)	-
Active disease—*n* (%)	18 (90)	-
In Remission—*n* (%)	2 (10)	-
With Immunosuppression	1/2 (50)	-
Without Immunosuppression	1/2 (50)	-

## Data Availability

The authors confirm that the data supporting the findings of this study are available within the article. Raw data were generated at the Department of Neurology with Institute of Translational Neurology, University Hospital Münster. The data presented in this study are available on request from the corresponding author [J.D.L.].

## Supplementary Materials

The following are available online at https://www.mdpi.com/article/10.3390/cells10051139/s1, Table S1. Clinical vignettes of patients with PACNS.
