# Supplementary material for: Profiling Complement System Components in Primary CNS Vasculitis
Source: Cells. 2021 May 8;10(5):1139. doi: 10.3390/cells10051139 (PMC8150982; doi:10.3390/cells10051139)
Supplement: Supplementary file 1 [file cells-10-01139-s001.zip › cells-1180911-supplementary.pdf]

Supplementary File

Table S1. Clinical vignettes of patients with PACNS

Pat	Age (yrs.)	Symptoms	MRI/DBI	CSF	DSA/ MRA	Biopsy	Disease course	Immunosuppressive Treatment*
1	33	Dizziness, hemianopsia, left-sided paresthesia	Multifocal ischemic stroke lesions DBI: pos	WBC 3/3 /µl, Pro: 350 mg/l OCB: neg., Cyto: N.p.	Multifocal irregular alternating stenosis/ occlusions and dilatations	N.p.	Active (Onset)	None
2	41	Headache, seizures, left-sided hemi-paresis	Contrast-enhanced lesion of the frontal lobe DBI: N.p.	WBC 2/3 /µl, Pro: 643 mg/l, OCB: neg, Cyto: No pathologies	No pathologies	Pos.	Active (Onset)	None
3	26	Headache, left-sided armparesis	Multifocal ischemic stroke lesions DBI: neg.	WBC 64/3 /µl, Pro: 324 mg/l, OCB: neg., Cyto: lympho-monocytic pleocytosis	Multifocal irregular alternating stenosis/ occlusions and dilatations	N.p.	Active (Onset)	None
4	59	Right-sided hemiparesis, dysarthria	Multifocal ischemic stroke lesions DBI: pos	WBC 32/3, Pro: 400mg/l intrathecal IgM>IgA-synthesis, OCB: neg., Cyto: lymphomonocytic pleocytosis,	Multifocal irregular alternating stenosis/ occlusions and dilatations	N.p.	Active (Onset)	None
5	26	Left-sided hemi-paresis	New ischemic stroke on the right side DBI: pos	WBC 4/3 /µl, Pro: 258 mg/l OCB: neg., Cyto: No pathologies	Progressive multifocal irregular alternating stenosis/ occlusions and dilatations	N.p.	Active (Relapse)	Cortisone, RTX
6	38	Headache, double vision, left-sided hemi-paresis	New ischemic stroke on the right side DBI: pos	WBC 6/3 /µl, Pro: 611 mg/l, OCB: neg., Cyto: No pathologies	Multifocal irregular alternating stenosis/ occlusions and dilatations	Pos.	Active (Onset)	None
7	55	Aphasia, dysarthria, facial palsy on the right side	New ischemic stroke on the left side	WBC 176/3 /µl, Pro: 584 mg/l, OCB: 2 isolated bands, identical	Progressive multifocal irregular alternating stenosis/	N.p.	Active (Relapse)	Cortisone, CYC, RTX

			DBI: neg.	bands, Cyto: lymphomonocytic pleocytosis	occlusions and dilatations			
8	55	Left-sided hemi-paresis	New ischemic stroke on the left side<br>DBI: N.p.	WBC 14/3 /µl, Pro: 352 mg/l, intrathecal IgM-synthesis, OCB: neg., Cyto: lymphomonocytic pleocytosis	Progressive multifocal irregular alternating stenosis/ occlusions and dilatations	Neg.	Active (Relapse)	Cortisone, CYC, MTX
9	47	Headache , right-sided hemiparesis	New ischemic stroke on the right side<br>DBI: Neg.	WBC 7/3 /µl, Pro: 452 mg/l OCB neg, Cyto: No pathologies	Progressive multifocal irregular alternating stenosis/ occlusions and dilatations	Neg.	Active (Relapse)	Cortisone, CYC, Aza
10	45	Double vision, dizziness, nausea, paresthesia on the right hand	New ischemic stroke in the brainstem<br>DBI: Neg.	WBC 120/3 /µl, 575 mg/l Prot, OCB: neg, Cyto: lymphomonocytic pleocytosis	Progressive multifocal irregular alternating stenosis/ occlusions and dilatations	Pos.	Active (Relapse)	Cortisone, CYC, MTX
11	43	Hemianopsia, dizziness, aphasia, progressive left-sided hemiparesis, seizures	Contrast-enhanced lesions<br>DBI: Neg.	WBC 2/3 /µl, Pro: 535 mg/l intrathecal IgA>IgM synthesis, isolated OCB Cyto: No pathologies	New stenosis of intracranial vessels, no vessel irregularities of brain vessels previously	Pos.	Active (Relapse)	Cortisone, Aza, CYC, RTX
12	47	Headache, dizziness, nausea, cognitive deficits	Multifocal ischemic stroke lesions<br>DBI: N.p.	WBC 76/3 /µl, Pro: 444 mg/l intrathecal IgG- synthesis, OCB: pos., Cyto: lymphomonocytic pleocytosis	No pathologies	N.p.	Active (Relapse)	Cortisone, MTX
13	69	Left-sided hemiparesis, cognitive deficits	New ischemic stroke on the right side<br>DBI: pos.	WBC 0 /µl, Pro: 522 mg/l OCB: neg., Cyto: No pathologies	Progressive multifocal irregular alternating stenosis/ occlusions and dilatations	N.p.	Active (Relapse)	Cortisone, further immunosuppressive therapy planned

14	49	Cognitve deficits, dyslexia	Contrast-enhanced lesions DBI: N.p.	WBC 6/3 /µl, Pro 798mg/l, intrathecal IgG- synthesis, isolated OCB Cyto: No pathologies	No pathologies	Pos.	Active (onset)	None
15	23	Headache, encephalopathy, seizures	Multiple contrast-enhanced lesions DBI: N.p.	WBC 360/3 /µl, Pro: 1614 mg/l, intrathecal IgM>IgA synthesis, OCB pos, Cyto: lymohomonocytic pleocytosis	No pathologies	Pos.	Active (onset)	None
16	36	Right-sided hemiparesis	Multifocal ischemic stroke lesions DBI: Pos.	WBC 4/3 /µl, Pro 597 mg/l, OCB: neg, Cyto: No pathologies	Multifocal irregular alternating stenosis/ occlusions and dilatations	Neg.	Active (onset)	None
17	31	Left-sided hemiparesis	Multifocal ischemic stroke lesions DBI: Pos.	WBC 0 /µl, Prot 280 mg/l, OCB: neg., intrathec. IgM-Syntehsis, Cyto: No pathologies	Multifocal irregular alternating stenosis/ occlusions and dilatations	N.p.	Active (onset)	None
18	44	Headache	New ischemic stroke on the right side DBI: pos.	WBC 1/3 /µl, 663mg/l, OCB neg., Cyto: No pathologies	Multifocal irregular alternating stenosis/ occlusions and dilatations	Neg.	Active (onset)	None
19	53	Headache, dizziness not due to vasculitis activity	No acute and new pathologies	WBC 1/3 /µl, Pro: 392 mg/l, OCB neg., Cyto: No pathologies	Multifocal irregular alternating stenosis/ occlusions and dilatations	N.p.	Inactive	None
20	61	Residual symptoms with encephalopathy, tremor, cognitive dysfunction No new, acute symptoms	No acute and new pathologies	WBC 0/3 /µl, Pro: 465 mg/l, Identical OCB, Cyto: No pathologies	Multifocal irregular alternating stenosis/ occlusions and dilatations	Neg.	Inactive	Cortisone, CYC, MTX

Abbreviations: MRI, magnetic resonance imaging; DBI, dark blood imaging; CSF, cerebrospinal fluid; DSA, digital subtraction angiography; MRA, MR angiography; WBC, white blood cell count; Pro, protein level in the CSF; OCB, oligoclonal bands; Cyto, Cytology; Pos, positive; Neg, negative; N.p., Not performed; CYC, cyclophosphamide; RTX, rituximab; MTX, methothrexate; Aza, azathioprine. * at time of blood draw, lumbar puncture
